# Design and experimental study on pruning machine of Yunnan edible rose

**DOI:** 10.1038/s41598-022-25551-1

**Published:** 2023-03-13

**Authors:** Huibin Zhu, Xu Zhang, Cheng Qian, Ran An, Haoran Zhao, Shiao Ma, Lizhen Bai

**Affiliations:** Faculty of Modern Agricultural Engineering, Kunming University of Science and Technology, Kunming, 650500 Yunnan Province China

**Keywords:** Engineering, Mechanical engineering

## Abstract

Edible rose is one of the main cash crops in Yunnan, China. Due to the high degree of lignification of rose stalks which is difficult to cut, and roses can only be pruned by hand after picking. Most of Yunnan Province is hilly landscape. Therefore, it is necessary to design an efficient small rose pruner for hills. Based on the experimental results of the physical and mechanical properties of rose stems, a simulation experiment of three different cutting methods was conducted to determine the optimal scheme. The results showed that single disc cutter is the best cutting method. An improved disc cutter with variable sliding angle is designed. In order to study the effects of the different structure parameters on the performance of the pruning machine and get the optimal combination of parameters, the orthogonal test was conducted. Forward speed, rotational speed of cutter, number of blades, cutting angle and knife-edge angle as factors, and the quality of pruning was selected as the evaluation index. The experiment results indicated that the working performance of machine was the highest when the number of blades was 6, rotational speed of cutter was 2000 rpm, cutting angle was 20°, knife-edge angle was 10°, forward speed was 0.5 m s^−1^. The average quality of pruning index, miss cutting index and tearing index were 90.6%, 5.68% and 3.72%, respectively. The designed machine can meet the agronomic requirements of Yunnan edible rose pruning operation. The research provides a reference for the design and extending of the pruning machine for edible rose.

## Introduction

Edible rose is one of the important economic crops in Yunnan of China. In 2021, its planting area was about 6000 hm^2^ and the economic benefit was nearly 740 million yuan^1^. In China, Edible roses are mainly used to make flower cakes, flower tea and other foods. Among the countries that grow roses on a large scale are Bulgaria, India, Turkey and France^2–4^. However, a small percentage of the roses planted in these countries are used for food processing and most of them are used to make rose oil. The continuous increase in the consumption of edible roses has brought considerable economic benefits to enterprises and flower farmers. At the same time, the labor costs have increased substantially. After the annual rose picking, the rose plants need to be pruned. The cultivation of edible rose in Yunnan is widely distributed and the terrain is complex. Besides, most of them are pruned in a manual way. Therefore there are many problems, such as the high labor intensity, unfavorable working environment, low efficiency of pruning and difficulty in recycling branches. At present, there is no machine dedicated to pruning roses. Large bush harvesters are often used in plain areas, and chain saws or garden scissors are often used in hilly and mountainous areas for pruning.

Outside China, the research of pruning machine started early and the technology was relatively mature^5–7^. More typical were the pruning machinery developed by France Kuhn s.a.s. company, the BRM series the pruning machinery designed by Rinier S.r.l. of Italy, the D600/D1200 pruning machinery produced by Jumar Agricola SL of Spain, and the Cristina Evo pruning machinery designed by Maschio Gaspardo S.p.A. of Italy. However, the above equipment is large and the cost is high, which makes it was difficult to adapt to the mountainous terrain of Yunnan Plateau.

At the beginning of the twenty-first century, some achievements have been made in the research of pruning machinery in China. The PJZ-1 type wine grape pruning machine with automatic obstacle avoidance system developed by Dong et al.^8^, and average efficiency was 0.05 hm^2^ h^−1^. The pea windrower equipment with TRIZ theory was designed by Li et al.^9^, and the height of cutting stubble was less than 40 mm and the harvesting efficiency reached 0.13–0.19 hm^2^ h^−1^. The Grape-picking DeVice with Grasping and Rotary-cut Type of Underactuated Double Fingered Hand designed by Yin et al.^10^, uses a disc knife to cut off the fruit stalk, which can realize flexible and nondestructive picking. The 3MDZ-4 self-propelled cotton topping and spraying combined machine designed by Peng et al.^11^, uses the lifting device to drive the disc knife to achieve precise topping, with functions of the seedling divided, the seedling holding, the topping and the pesticide spraying simultaneously. A planet gear driver of the reciprocating double-acting cutter was designed by Ran et al.^12^, the average power consumption of the planet gear driven double-acting cutter was 43.3% and 16.8% lower than that of the crank-linker driven single-acting cutter and double-acting cutter, respectively. The above machinery is not designed for rose stems and has not been validated in field trials in Yunnan rose plantations. Therefore, it cannot be directly applied to pruning edible roses in Yunnan Province.

The main methods used to cut shrub stalks are single disc cutter, double disc cutter and reciprocating cutter. In the current study only one cutting method is usually optimized and analyzed, so it is necessary to compare and analyze the cutting effects of three cutting methods. In the pre-design stage, simulation analysis is an efficient means. The premise of simulation analysis is to determine the physical and mechanical properties of rose stem, which is also missing in the existing research. In engineering problems, field tests are essential in order to obtain the key parameters of the edible rose pruning machinery.

Edible rose has great development prospects. Most edible roses in Yunnan, China are planted in hilly and mountainous areas. It usually takes about 150 h to manually prune one hectare of edible roses^13^. Studying a rose pruning machine that is suitable for working in small fields can greatly improve the production efficiency, which is of great significance to the development of the edible rose industry. This study measured the physical and mechanical properties of rose stem, completed the simulation experiments of three different cutting schemes, determined the best scheme, and improved and designed a single disc cutter with variable sliding cutting angle. Finally, an edible rose pruner suitable for hilly terrain in Yunnan was designed and field validation experiments were carried out.

## Material and methods

### Overall structure and working principle

**Figure 1:**
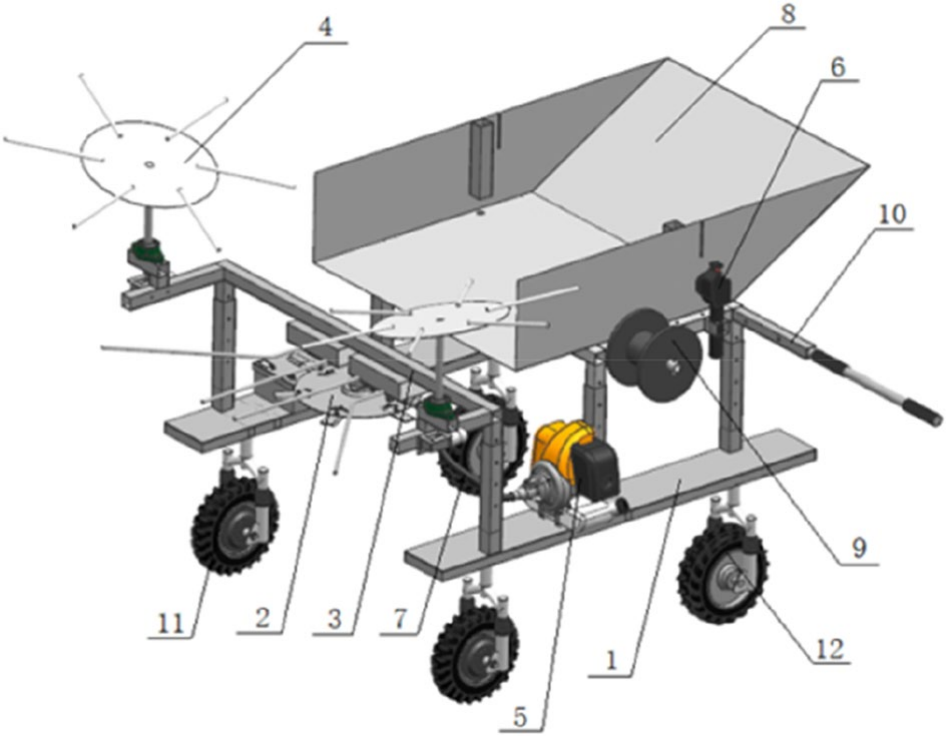
Schematic diagram of edible rose pruning machine. Note: 1, frame; 2, single disc cutter; 3, cutter support frame; 4, closing branches dish; 5, gasoline engine; 6, throttle controller; 7, flexible shaft; 8, collection box; 9, wire reel; 10, push rod; 11, directional wheel; 12, universal wheel.

A rose pruner with a single disc cutter device was designed to address the problems of dense rose plants and serious branch dispersal, and the high degree of lignification of branches that are not easy to cut, etc. The structure of the whole machine is shown in Fig. 1. The machine adopts the operation mode of cross-ridge pruning, and the worker can control the machine to advance in the field. Before the cutting process, the gasoline engine is started and the throttle controller is used to adjust the machine to the required cutting speed, and the cutting process is first passed through the branching process. The disc gathers the scattered rose branches on both sides to the middle, and the lever on the tray puts the branches into the branching rod, further bundles the stems of the plants, cuts the branches by a high-speed rotating cutter. The rose branches are fed into the recycling bin under the action of the dialing of the tray and the backward movement of the cutter head. The recycling box is laid in the recycling bin before the cutting operation. After the branches are filled with the recycling bins, the stems are bundled and recovered using the tying line, and the next stem cutting operation is performed.

### Physical and mechanical properties of rose stems

#### Collection of test samples

In Yunnan, China, edible roses are cut and grafted after two years of planting. The new stems begin to age after three years of growth. The flowering period of roses is in May and June each year. The rose variety selected in this study was "Dianhong Rose". The edible roses collected for the test were four years old. The test sample was the stem that has been pruned and has grown for one year. The test sample was collected in July. The test sample selected fresh plants that have been picked flowers and have healthy growth, no pests and no mechanical damage. The "five point sampling method" is used to collect the test samples of rose stems, that is, find two diagonal lines in the square test area, and the intersection of the two diagonal lines is taken as a sampling point. Then, take the other four points on the two diagonal lines where the distance from the four vertices is about a quarter of the length of the diagonal line as the sampling point for sampling.

#### Determination of morphological characteristics of rose plants

Two strains of pest-free and well-grown roses were randomly selected in the five sampling areas, and the height of the whole plant, the number of branches, the diameter of the bottom section (0–50 cm), the diameter of the middle section (50–100 cm) and the diameter of the top section (100–150 cm) were measured. The average height of the rose plants was 165.6 cm, the average number of branches was 5, the average diameter of the rose bottom, rose midpoint and rose top was 7.52 mm, 6.24 mm and 4.64 mm respectively (The experimental datasets can be obtained from the supplementary information Table 4).

#### Determination of rose stem density

According to the test method of density determination, the density of 10 groups of rose stems was measured, and the stems were weighed by JE502 electronic scale (The maximum weighing value is 500 g, and the minimum scale value is 0.01 g). As the rose stems are irregular samples, First, 100 ml of water was poured into a measuring cylinder with a volume of 250 ml, then the rose stems were completely pressed into the water with a fine needle and the volume difference was recorded, the measurement was repeated 10 times continuously. The volume is measured by drainage method, and then calculated the rose stem density according to formula (1). According to the analysis, the average density of rose stems was 0.344 g cm^−3 ^(The experimental datasets can be obtained from the supplementary information Table 4).

1 $$\rho { = }\frac{M}{V}$$

where $\rho$ is rose stem density, g cm^−3^; *M* is stem mass, g; *V* is stem volume, ${\mathrm{cm}}^{3}$.

#### Determination of mechanical properties of rose stem

The rose stems were cut, disbranched, cleaned and numbered. The test instrument was ZQ-700 series electric tensile and pressure testing machine developed by Zhituo Precision Instrument Co., Ltd. in Dongguan, China. The force resolution is 0.001 N, the force measurement accuracy is 1%, and the displacement control accuracy is 0.3%. The tensile, shear and bending tests were completed respectively with the corresponding clamps.

The measured tensile strength of rose stem is 12.346 MPa, the strain is 0.017, the elastic modulus is 677.847 MPa, the shear strength is 8.944 MPa, the flexural strength is 28.131 MPa, and the fracture deflection is 14.67 mm (The experimental datasets can be obtained from the supplementary information Table 4).

### Cutting scheme

As the core process of the edible rose pruning, the cutting mechanism plays a decisive role in the rose branch pruning. At present, the main types of cutters are disc rotary cutter and reciprocating cutter^14–16^. For the pruning operation of edible rose stems, three cutting schemes of single disc cutter cutting, double disc cutter cutting and reciprocating cutting were proposed. They were simulated by finite element pre-processing software Hypermesh developed by Altair and dynamics analysis software LS-DYNA developed by LSTC^17–19^. The resistance of the cutting tool during cutting and the throwing of the rose stem were analyzed, so as to choose a better cutting solution that meets the agronomic requirements.

#### Model meshing and related parameter definition

The geometric model was imported into Hypermesh as STP file and meshed (based on the feasibility, the rose stem model uses the single plant model). According to the measured data of physical properties and mechanical properties of rose stem, the parameters of density, elastic modulus, Poisson's ratio, tensile strength, shear strength and flexural strength of rose stem were defined in software. The tool material was defined as 45 steel, and the yield strength was 3.55 × 10^2^ MPa. The rotational speed of the disc cutter was 1500 rpm, the linear speed of the reciprocating cutter was 1.2 m s^−1^, and the feed speed was 0.8 m s^−1^. The contact type of tool model and rose stem model was CONTACT ERODING SURFACE TO SURFACE in the software. The simulation solution time was 0.5 s and the step time was 0.001 s. The data file of simulation was exported as file and imported into LS-DYNA for repeated solution for three times. Then import the d3plot file generated after the solution into LS-DYNA post-processing plug-in LS PrePost for analysis^20^.

#### Simulation process

**Figure 2:**
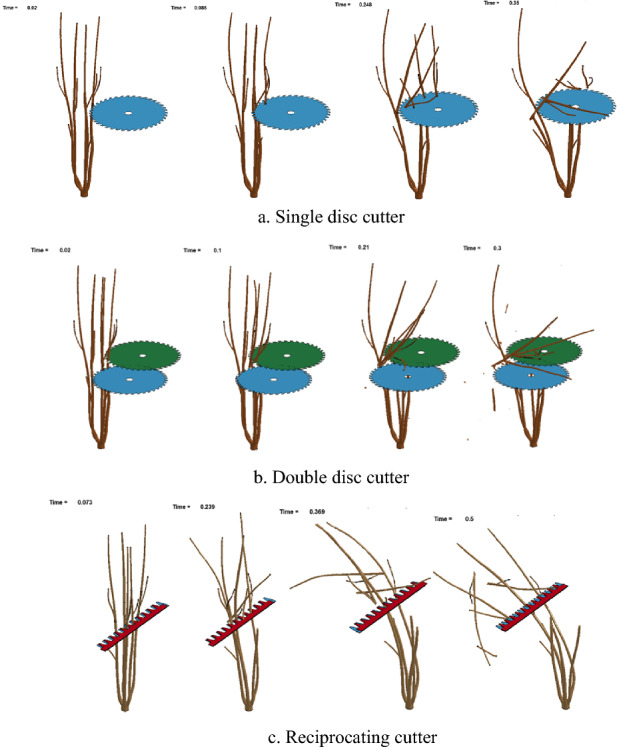
The process of cutting rose stem.

The process of cutting rose stem with three different cutting tools is shown in Fig. 2. The shearing condition of stems and the movement direction of the rose branches after cutting could be observed. The images showed that the rose branches cut by the single disc cutter were relatively complete without broken branches, and backward branches were favorable to collect. The double disc cutting mode was more likely to make branches fragmented and scattered everywhere, which was not conducive to gathering. The rose branches cut by the reciprocating cutter mostly fall along the direction of the cutter, which was inconvenient to gathering also.

Cutting power consumption and force uniformity are the most important quantitative evaluation indicators^21^. Cutting power consumption and cutting resistance are in direct proportion. Variation coefficient of cutting resistance was used to characterize the force uniformity. The maximum equivalent stress, maximum cutting resistance, average cutting resistance, variation coefficient of cutting resistance of single disc, double disc and reciprocating cutter were obtained as shown in the Table 1 (The experimental datasets can be obtained from supplementary information 1–3). It can be seen that the maximum equivalent stress was less than 355 Mpa, and the tool will not be damaged. The maximum cutting resistance, average cutting resistance and variation coefficient of cutting resistance of single disc cutter were the minimum. It shows that the cutting power consumption and force uniformity of single disc cutter were the minimum, and the rose stalks cut by single disc cutter were the easiest to recycle, Based on the above data, using a single disc knife cutter is the best scheme.

**Table 1:** Simulation output.

Parameter	Single disc cutte	Double disc cutter	Reciprocating cutter
Maximum equivalent stress/MPa	59.91	69.98	67.76
Maximum cutting resistance/N	468.16	1090.00	558.33
Average cutting resistance/N	33.33	119.75	55.03
Variation coefficient of cutting resistance/%	116.98	135.64	139.34

### Cutting mechanism design

**Figure 3:**
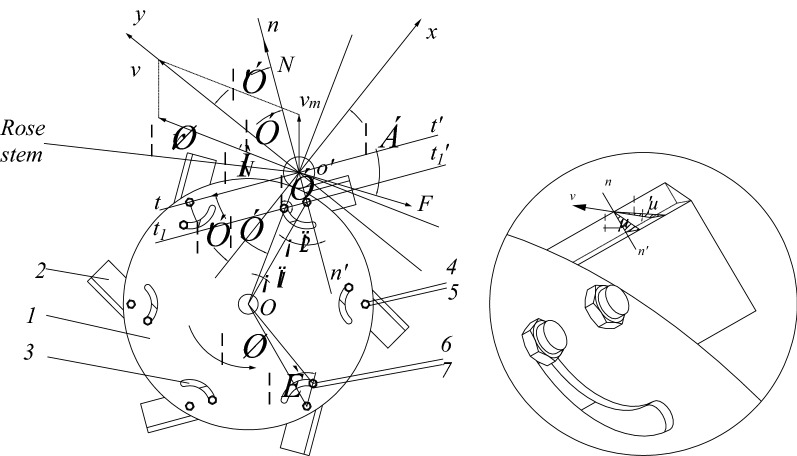
Improved disc cutter. Note: 1, cutter head; 2, blade; 3, Bolt A; 4, Nut A; 5, Bolt B; 6, Nut B; 7, chute.

It is known that the stalk cutting method of the common saw disc cutter is sliding cutting^22^. Based on the ordinary disc cutter, a disc cutter with variable sliding cutting Angle was designed in this study (as shown in the Fig. 3). By changing the positions of bolt B and nut B in the chute, the static sliding cutting Angle τ and dynamic sliding cutting Angle τ’ of the blade can be changed. The right-angle coordinate system *yo 'x* is established (Fig. 3). The *t−t '*and *t*_*1*_*−t*_*1*_*'* are parallel lines of the tangent lines of the blade. The *n–n'* line is the normal of the blade line. The *o–o'* line is the line between the center of the cutter and the center of the rose stem. The ωr line is perpendicular to *o–o'.*

The instantaneous balance equation of blade cutting rose stem is as follows:

2 $$\begin{array}{c}\left\{\begin{array}{c}N\mathrm{sin}{\tau }^{^{\prime}}+{F}_{1}\mathrm{cos}\alpha -\mu N\mathrm{cos}{\tau }^{^{\prime}}=0\\ N\mathrm{cos}{\tau }^{^{\prime}}-{F}_{1}\mathrm{sin}\alpha +\mu N\mathrm{sin}{\tau }^{^{\prime}}=0\end{array}\right.\end{array}$$

Derivation leads to:

3 $$\begin{array}{c}N=\frac{{F}_{1}\left(\mathrm{sin}\alpha -\mathrm{cos}\alpha \right)}{\left(1+\mu \right)\mathrm{sin}{\tau }^{^{\prime}}+\left(1-\mu \right)\mathrm{cos}{\tau }^{^{\prime}}}\end{array}$$

where, *v*_*m*_ is the forward speed of the machine; ω is the angular speed of the cutter head rotation; r is the distance of *o–o'*; *v* is the absolute speed of the blade; τ is static sliding cutting Angle; τ' is the dynamic sliding cutting Angle; *N* is cutting force; *μN* is friction force; *F* is the resultant force of other forces on the rose stem; α is the Angle between F and ox.

*N* is a key factor affecting cutting quality^23^. It can be seen from the formula that N is related to τ', so the sliding cutting Angle τ' is an important parameter affecting the cutting effect, that is, the blade cutting Angle ∠2 is a key parameter for cutting rose stems. And because the vector equation of absolute velocity is:

4 $$\begin{array}{c}\underset{v}{\to }=\underset{\omega r}{\to }+\underset{{v}_{m}}{\to }\end{array}$$

Therefore, *ω* and *v*_*m*_ are also key parameters for cutting rose stalks. In addition, it can be seen from the Fig. 3 that the blade Angle *Φ* is also a key parameter affecting the cutting effect. In addition, the number of blades and blade length of the improved disc cutter are different from that of the ordinary disc cutter.

1. Diameter of cutter head (D): The diameter of the disc cutter is usually in the range of 150–1500 mm. For wood cutting materials such as solid wood or shrub, the outer diameter is usually from 350 to 450 mm. The rose plant belongs to the shrub, so the diameter of the cutting disc is designed as 350 mm.
2. Thickness of cutting disc (T): The thickness of the cutting disc is usually related to the diameter of the cutting disc, as shown in the formula (5):
  5 $$T = K \cdot \sqrt D$$
  where *T* is thickness of cutting disc, mm; *D* is diameter of cutting disc, mm; *K* is coefficient of cutter thickness, K is 0.065 when diameter of cutting disc in the range of 150–650 mm^24^.

According to the formula (5), when the diameter of the cutting disk is 350 mm and the value of K is 0.065, the thickness S of the cutter head is 1.2 mm.

3. Number of blades (Z): During the cutting process, the cutter-head performs linear motion and rotary motion at the same time. The motion analysis of the upper edge shows that the trajectory is trochoid, and the trochoid band are composed of trochoid of different cutting edges. The trochoid band width is approximately equal to the length L of the blade. Under the premise of no missing cut, the sum of the bandwidths of all the residual pendulum bands in the forward direction of the whole machine should be greater than or equal to the forward distance of the whole machine^25^. The formula for calculating the number of blades is as follows:
  6 $$\begin{array}{c}Z\cdot L\ge \frac{2\pi {v}_{m}}{\omega }\end{array}$$
  7 $$\begin{array}{c}Z\ge \frac{2\pi {v}_{m}}{L\omega }\end{array}$$
  where *Z* is number of blades, *piece*; *v*_*m*_ is forward speed of machine, m/s; *L* is blade length, m; *ω* is cutter angular velocity, ω = 2πn/60 in rad/s.

Combined with the structure size of the cutter head, the maximum number of blades is determined to be 6.

4. Blade length (L): During the pruning of the rose branches, it may occur that the cutter-head collides and bends the stem. When one of the cutting edges fails to completely cut the rose stem and requires other cutting edges to make the cutting, the cutter head contacts the stem in advance, resulting in the stem not being cut off completely and bend it. The problem arises from the length L of the blade. When the blade length L is too short, it is easy to cause the blade to collide with the stem and affect the quality of the pruning. In order to avoid this, the blade length L should satisfy:
  8 $$L\cos \varphi \ge d + \frac{{2\pi v_{m} }}{Z\omega }$$
  where *φ* is cutter-head knife-edge angle, °; *d* is rose stem diameter, m.

When the angle of the cutter head is zero, the diameter D of the rose stem is 6 mm, the forward speed of the whole machine is 1 m s^−1^, and the angular velocity of the cutter head is 25 rad s^−1^, the length of the designed blade should be longer than 0.048 m based on the formula (8). And according to the agronomic requirements of rose pruning, the rose stem should be pruned at a distance of 50 cm from the ground. The branch divergence measurement of the rose plant at the shearing site shows that the maximum dispersion diameter of the branches is 550 mm. Through the branching mechanism of the pruning machine, the outer diameter of the dispersion can be folded from 550 to 400 mm, the diameter of the cutting disc is 350 mm, the length of the cutting edge is designed to be 50 mm, and the cutting width is 450 mm, which meets the pruning requirements.

5. Cutting angle *θ*: The sliding cutting Angle t can be changed by changing the cutting Angle θ. It can be seen from the Fig. 3:
  9 $$\begin{array}{c}\theta =\angle 2=\tau -\angle 1\approx \tau -10^\circ \end{array}$$

It is known that the optimal value of t ranges from 0° to 40°^22,26–28^, so the range of *θ* ranges from 0° to 30°.

6. Blade knife-edge angle *Φ*: As the knife-edge angle of the cutting edge increases, the cutting power consumption increase. Instead, the wear resistance of cutter will decrease. By reviewing the domestic scholars' design analysis of the botanic stem cutters, the blade knife-edge angle is between 5° and 30°^26,29,30^. Therefore, with reference to the previous mechanical properties test of rose stems and other stem cutters, three different blade angles are designed, which are 10°, 15° and 20° respectively.

### Design of field orthogonal experiment

**Figure 4:**
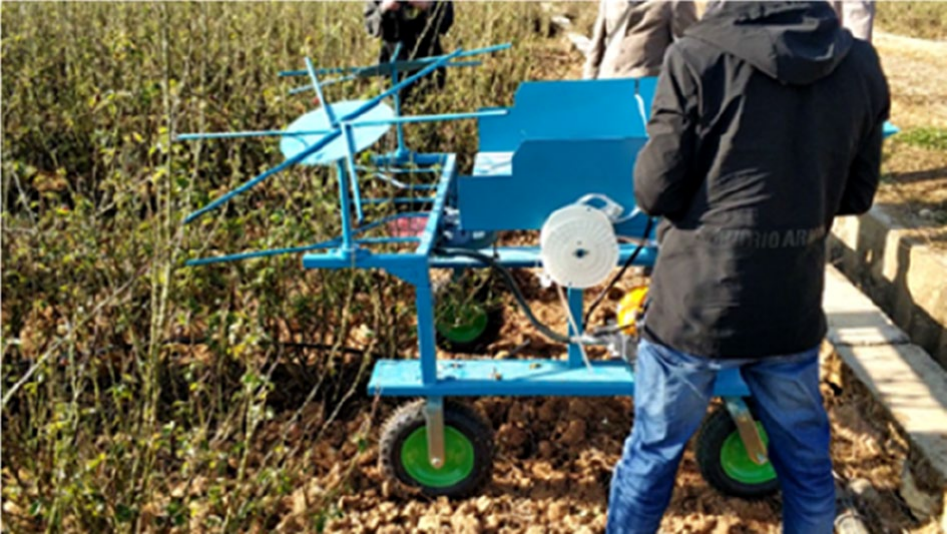
Field cutting experiment.

The experiment was conducted in the Jiahua edible rose organic planting base in Qujing City, Yunnan Province, China. The experiment process was shown in Fig. 4. In order to study effects of the different structure parameters on the performance of the pruning machine of edible rose, forward speed, rotational speed of cutter, number of blades, cutting angle and knife-edge angle were chose as the experimental factors, and quality of pruning index as the evaluation indexes, to conduct the orthogonal experiment^27,28^. According to the operating parameters of shrub pruning machinery, the parameter range of the rose pruning machine was determined as follows: the forward speed was 0.25–0.75 m s^−1^, the rotate speed of the cutter was 1000– 2000 rpm, the number of blades was ≤ 6, the cutting angle was 10°–30°, and the knife-edge angle was 10°–20°.Each factor concludes 3 levels, the orthogonal experiment was performed by L_18_ (3^7^) orthogonal table. Randomly select the fifth and seventh columns as the control columns for comparison. The experiment factor level is shown in Table 2. Operation area of machine was a field of 10 m ridge length. After experiment, select the starting, middle and end of the cutting path as the sampling points, and select 5 rose bushes at each sampling point for statistics of the qualified index of pruning.

**Table 2:** Experiment factor level.

Level	Factor				
	A:Forward speed /(m s^−1^)	B:rotate speed of the cutter /(rpm)	C:number of blades	D:cutting angle *θ* /(°)	E:knife-edge angle *Φ*/(°)
1	0.25	1000	2	10	10
2	0.5	1500	3	20	15
3	0.75	2000	6	30	20

After shearing, if there is no missing cutting or tearing, the rose branch is qualified for cutting. The calculation formula of the rose branch pruning rate is:

10 $$\upeta = \frac{{\sum {N - \left( {\sum S + \sum P } \right)} }}{\sum N }$$

where*η*is pruning pass rate, *%*; *N* is total number of branches; *S* is total number of tearing branches; *P* is total number of missing branches.

**Figure 5:**
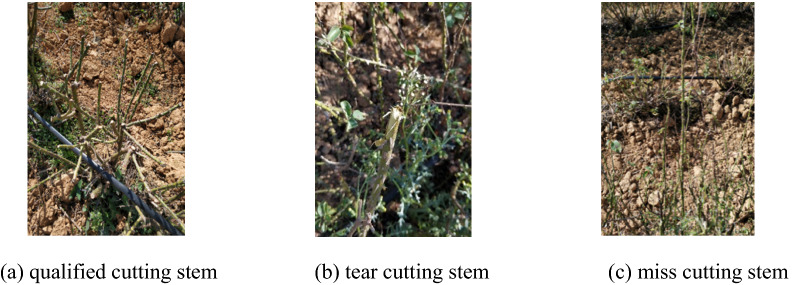
Rose branch status after cutting.

The state of the branches after the shearing by the rose pruning machine is shown in Fig. 5. The cut of the rose branches is smooth, the tingling without tear and the rupture of the stem are qualified for cutting. The tearing phenomenon is mainly due to the pulling, re-cutting, tool collision. Tearing damages the rose plants and affect their growth. Missing the branches will increase the labor of the workers. Therefore, the phenomenon of tearing and missing of the branches should be avoided.

### Verification test

The field experiment was carried out on the rose pruning machine with the optimal combination of parameters. To explore the final operation quality of the designed machine. The test method is the same as Part 2.5. The Miss cutting index Y_1_ and tearing index Y_2_ are as follows:

11 $$\begin{array}{c}{Y}_{1}=\frac{\sum L}{\sum Z}\times 100\%\end{array}$$

12 $$\begin{array}{c}{Y}_{2}=\frac{\sum S}{\sum Z}\times 100\%\end{array}$$

### Ethical statement

We declare that all experimental studies on cultivated edible roses in this study, including the collection of plant materials, comply with the guidelines and legislation of relevant institutions, China and the world. The edible rose used in this study is not an endangered species. We declare that the plant samples used in the experiment are collected from private land and have obtained the permission of the land owner.

## Results and discussion

### Analysis of field orthogonal experiment results

The experiment results are shown in Table 3 (The experimental datasets can be obtained from the supplementary information Table 4). The analysis of the range method shows that the order of the cutting quality factors in the process of cutting is: number of blades, cutting speed, cutting angle, blade knife-edge angle and forward speed. Comparing the value of k_i_, it can be seen that the optimal parameter combination for the rose pruning machine operation is C_3_B_3_D_2_E_1_A_2_, that is, the number of blades was 6, rotational speed of cutter was 2000 rpm, cutting angle was 20°, knife-edge angle 10° and forward speed was 0.5 m s^−1^.

**Table 3:** Analysis of orthogonal experiment results in the field.

Experiment number	Factors							Quality of pruning index η/%
	A	B	C	D	Vacant column	E	Vacant column	
1	1	1	1	1	1	1	1	79.3
2	1	2	2	2	2	2	2	90.5
3	1	3	3	3	3	3	3	86.1
4	2	1	1	2	2	3	3	77.5
5	2	2	2	3	3	1	1	84.7
6	2	3	3	1	1	2	2	87.1
7	3	1	2	1	3	2	3	81.1
8	3	2	3	2	1	3	1	86.5
9	3	3	1	3	2	1	2	82.1
10	1	1	3	3	2	2	1	81.7
11	1	2	1	1	3	3	2	78.2
12	1	3	2	2	1	1	3	89.7
13	2	1	2	3	1	3	2	87.1
14	2	2	3	1	2	1	3	92.3
15	2	3	1	2	3	2	1	89.4
16	3	1	3	2	3	1	2	91.8
17	3	2	1	3	1	2	3	83.4
18	3	3	2	1	2	3	1	88.6
K_1_	505.5	498.5	489.9	506.6	513.1	519.9	510.2	
K_2_	518.1	515.6	521.7	525.4	512.7	513.2	516.8	
K_3_	513.5	523	525.5	505.1	511.3	504	510.1	
k_1_	84.25	83.08	81.65	84.43	85.52	86.65	85.03	
k_2_	86.36	85.93	86.95	87.57	85.45	85.53	86.13	
k_3_	85.6	87.17	87.58	84.18	85.22	84	85.01	
R	2.11	4.09	5.93	3.39	0.3	2.65	0.12	
The order of factors	C B D E A							
Optimal combination	C_3_B_3_D_2_E_1_A_2_							

In a column, K_i_ is equal to the sum of η in the row with factor level i; ${k}_{i}=\frac{{K}_{i}}{s}$(s is the level number of each factor, s = 3); R is the range, $R=max\left\{{k}_{1},{k}_{2},{k}_{3}\right\}-min\left\{{k}_{1},{k}_{2},{k}_{3}\right\}$.

### Verification test

The experiment results are shown in Table 4. The average quality index, miss cutting index and tearing index were 90.6%, 5.68% and 3.72%, and their coefficient of variation were 10.21**,** 12.37 and 1.14, respectively. These results verify the correctness and reliability of the orthogonal test, as well as the feasibility of the machine.

**Table 4:** Optimal case experiment results analysis.

Number	Miss cutting index Y_1_/%	Tearing index Y_2_/%	Quality index η/%
1	6.5	4.3	89.2
2	5.6	3.8	90.6
3	6.1	4.1	89.8
4	4.8	3.1	92.1
5	5.4	3.3	91.3
Average	5.68	3.72	90.6
Standard deviation	0.58	0.46	1.03
Coefficient of variation/%	10.21	12.37	1.14

### Discussion

According to the statistics, the quality index of all kinds of pruning machines is 80–95%, the missed cutting index is 3–10%, and the tear index is 2–8%^8–12^. The qualified index of the rose pruning machine designed in this research was 90.6%, the missed cutting index was 5.68%, and the tear index was 3.72%. The operation effect were excellent. The size of the machine designed was 1200 mm * 110 mm * 1210 mm, which was more suitable for the field terrain of Yunnan, China, compared with the large combined pruning machines. The working efficiency can reach 700m^2^/h, which is more than 10 times higher than that of manual pruning.

## Conclusion

The edible roses in Yunnan, China are planted in hilly and mountainous areas, and only the stems of roses can be manually pruned every year. The rose stem is not easy to cut, and the efficiency of manual pruning is low and the quality is poor. Therefore, it is of great significance to solve the problem of mechanized pruning of rose stems in Yunnan, China, to improve economic benefits and production efficiency. In this research, the physical and mechanical properties of rose stems were measured, simulation analysis of three different cutting solutions was conducted by Hypermesh and LS-DYNA to come up with the best cutting solution, and an improved disc cutter with variable slip cutting angle was designed. The optimal parameters of the machine were obtained through orthogonal tests. Finally, validation tests were conducted. A small hand-pushed rose pruner that integrates gathering, cutting and baling for recycling was finally developed.

1. The physical and mechanical properties of rose stems showed that the average height of rose plants was 165.6 cm and the density was 0.344 g cm^−3^. The strain of rose stem was 0.017, fracture deflection was 14.67 mm. The tensile strength, elastic modulus, shear strength and bending strength were 12.346, 677.847, 8.944 and 28.131 MPa.
2. Three cutting schemes (single disc cutter, double disc cutter and reciprocating cutter) were analyzed by Hypermesh finite element pre-processing software and LS-DYNA dynamics analysis software. The rose branches cut by the single disc cutter were relatively complete without broken branches, and backward branches were favorable to collect. The cutting model of double disc made many branches fragment and be scattered everywhere. The rose branches cut by the reciprocating cutter mostly fall along the direction of the cutter. Rose stalks cut by Single disc cutte are the easiest to recycle. The maximum equivalent stress, maximum cutting resistance, average cutting resistance and variation coefficient of cutting resistance of single disc cutter are the smallest, with values of 59.91 Mpa, 468.16 N, 102.15 N and 15.28% respectively. It shows that the single disc cutter has the smallest cutting power consumption, the most uniform force and will not be destroyed.
3. An edible rose pruning machine was designed by analyzing the cutter disc diameter, cutter thickness, bore diameter, number of cutting edges, cutting edge length, cutting angle and knife-edge angle of the single disc cutter. The field orthogonal experiment was carried out on the rose pruning machine. The optimal combination of the parameters was that the number of blades was 6, rotational speed of cutter was 2000 rpm, cutting angle was 20°, knife-edge angle 10° and forward speed was 0.5 m s^−1^. Field tests have shown that our machines are suitable for dense plantations. At this time, the average quality index, miss cutting index and tearing index were 90.6%, 5.68% and 3.72%.

This study fills the gap of mechanical pruning of edible roses in Hilly and mountainous areas of Yunnan Province, China. The edible rose pruning machine designed meets the requirements of the rose pruning operation in Yunnan Province, China and can improve the agricultural production efficiency. This study can provide reference for the design and improvement of shrub pruning machinery working in Hilly and mountainous areas. In this study, only specific varieties of roses were tested at this stage. However, there are many varieties of roses in China with different physical characteristics. Therefore, the designed machine is only suitable for Rosa dianhong in China at present. It is still necessary to verify the suitability of the machine for different varieties at the next stage. Our research is replicable, in the future, the same test method in this study can be used to obtain the machine parameters required for cutting different rose stems. It can also study the influence of mechanical pruning on the growth of edible roses and improve the cutting scheme.

## Supplementary Information

Supplementary Information 1.

Supplementary Information 2.

Supplementary Information 3.

Supplementary Table 4.

## Data Availability

The analyzed datasets are available from the corresponding author on reasonable request.
