# Supplementary material for: Design and experimental study on pruning machine of Yunnan edible rose
Source: Sci Rep. 2023 Mar 13;13:4118. doi: 10.1038/s41598-022-25551-1 (PMC10011599; doi:10.1038/s41598-022-25551-1)
Supplement: Supplementary file 4 — Supplementary Table 4. [file 41598_2022_25551_MOESM4_ESM.pdf]

Supplementary material：

Tab 1 Rose morphological measurement

Sample number	height/cm	Number of branches	Bottom diameter/mm	Middle diameter /mm	Top diameter/mm
1	156	5	7.45	6.26	4.73
2	176	6	7.36	5.98	4.45
3	172	4	8.18	6.42	5.20
4	168	6	7.62	6.38	4.16
5	165	3	7.24	6.49	4.42
6	175	5	7.05	6.34	4.61
7	157	7	7.69	6.02	4.67
8	166	5	8.02	6.18	4.37
9	162	4	7.51	5.89	4.96
10	159	6	7.12	6.40	4.82
average value	165.6	5	7.52	6.24	4.64

Tab 2 Density measurement results of rose stalks

Sample number	quality（g）	volume（cm^3）	density(g/cm^3)
1	6.54	20.3	0.322
2	7.32	18.7	0.392
3	6.98	20.1	0.347
4	7.64	25.6	0.298
5	6.88	19.3	0.356
6	8.15	26.5	0.307
7	7.53	20.9	0.361
8	6.97	18.8	0.371
9	7.28	23.1	0.315
10	6.37	17.1	0.373
average value			0.344

Tab 3 Measurement Data of Mechanical Properties of Rose Stalks

Sample	tensile strength/MPa	strain	elastic modulus/MPa	shear strength/MPa	flexural strength/MPa	fracture deflection/mm
1	11.624	0.017	666.132	11.183	35.356	12.691
2	14.316	0.017	823.469	8.880	32.428	13.238
3	12.844	0.019	681.379	9.102	28.354	15.165
4	10.025	0.012	811.741	7.070	26.409	15.757
5	9.368	0.016	591.228	8.071	26.315	14.784
6	9.508	0.012	805.763	8.692	27.407	17.419
7	10.689	0.016	668.063	9.054	25.287	14.982
8	10.866	0.021	517.429	9.154	28.991	12.350
9	10.172	0.019	535.368	9.293	22.630	15.692

max	16.12	0.021	823.469	11.183	35.356	17.419
min	9.368	0.012	517.429	7.070	19.218	12.350
average	12.346	0.017	677.847	8.944	28.131	14.675

Tab 4 Quality of pruning index

Experiment number	1	2	Rejected data	Average Quality of pruning index/%
1	79.5	79.1		79.3
2	90.1	90.9		90.5
3	85.7	86.5	83.3	86.1
4	77.5	77.5	82.7	77.5
5	84.5	84.9		84.7
6	87.0	87.2		87.1
7	80.8	81.4	75.0	81.1
8	85.7	87.3		86.5
9	82.1	82.1		82.1
10	81.6	81.8		81.7
11	77.9	78.5		78.2
12	89.5	89.9		89.7
13	87.1	87.1		87.1
14	92.3	92.3	78.8	92.3
15	89.4	89.4		89.4
16	91.3	92.3		91.8
17	83.1	83.7		83.4
18	88.3	88.9		88.6
